# Sandwich-like Ni_2_P nanoarray/nitrogen-doped graphene nanoarchitecture as a high-performance anode for sodium and lithium ion batteries

**DOI:** 10.1016/j.dib.2018.08.158

**Published:** 2018-08-30

**Authors:** Caifu Dong, Lijun Guo, Yanyan He, Chaoji Chen, Yitai Qian, Yanan Chen, Liqiang Xu

**Affiliations:** a School of Chemistry and Chemical Engineering, Shandong University, Jinan 250100, China; b State Key Laboratory of Coordination Chemistry, Nanjing University, Nanjing 210093, China; c School of Electrical and Electronic Engineering, Huazhong University of Science and Technology, Wuhan 430074, China; d School of Life Sciences, Tsinghua University, Beijing 100084, China

## Abstract

The data presented in this article are related to the research article entitled “Sandwich-like Ni_2_P Nanoarray/Nitrogen-Doped Graphene Nanoarchitecture as a High-Performance Anode for Sodium and Lithium Ion Batteries (Dong et al., 2018)”. This work shows the morphology and structural of Ni_2_P/NG/Ni_2_P and the electrochemial performance of Ni_2_P/NG/Ni_2_P.

**Specifications table**

**Table:** 

Subject area	*Chemistry*
More specific subject area	*Inorganic chemistry*
Type of data	*Figures*
How data was acquired	*Using SEM, TEM, FT-IR, XRD*
Data format	*Raw and analyzed data*
Experimental factors	*Powder samples*
Experimental features	*Date illustrate the morphology and structural of Ni*_*2*_*P/NG/Ni*_*2*_*P*
Data source location	*Jinan, China*
Data accessibility	*C. Wu, P. Kopold, P. A. V. Aken, J. Maier, Y. Yu, High Performance Graphene/Ni*_*2*_*P Hybrid Anodes for Lithium and Sodium Storage through 3D Yolk–Shell-Like Nanostructural Design, Adv. Mater. 29 (2017) 1604015.**[1]*

**Value of the data**

• Relevant data on the morphology and structural of Ni_2_P/NG/Ni_2_P.
• Data to be used on understanding the structure-reactivity correlations.
• These data provide electrochemical performance of Ni_2_P/NG/Ni_2_P composite as anode material in SIBs.

## 1 Data

The data presented in this manuscript have been generated in a study searching for a novel Ni_2_P/NG/Ni_2_P material via solvothermal method and phosphorization treatment. The successful fabrication of Ni_2_P/NG/Ni_2_P was confirmed by the SEM, TEM, HRTEM and XRD results. Excellent electrochemical performance of SIBs batteries was obtained for the Ni_2_P/NG/Ni_2_P composite (Fig. 3).

## 2 Experimental design, materials and methods

### 2.1 Synthesis of Ni-based precursor/GO/Ni-based precursor nanoarrays

**Fig. 1:**
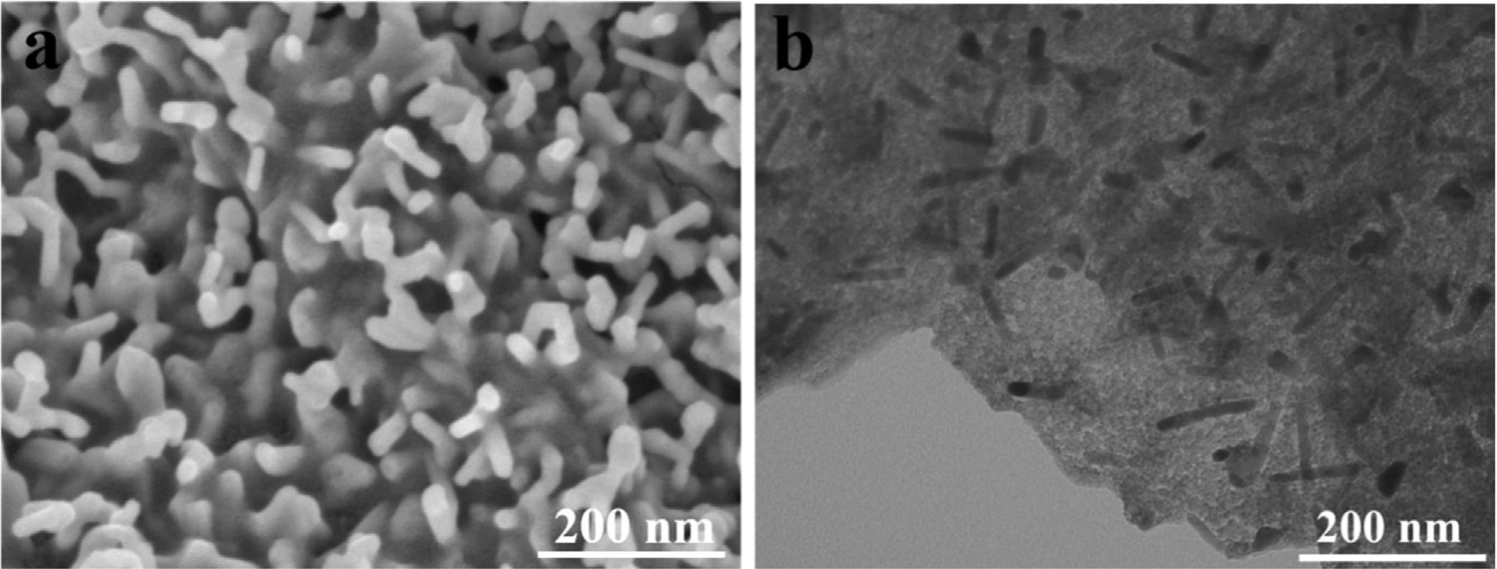
(a) SEM and (b) TEM image of Ni_2_P/NG/Ni_2_P.

**Fig. 2:**
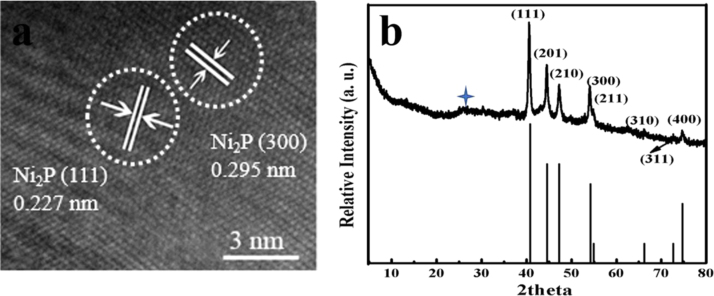
(a) HRTEM and (b) XRD of Ni_2_P/NG/Ni_2_P.

In a typical synthesis, GO (3.0 mg) was dispersed in the solvents of 1 mL methanol (CH_3_OH) and 7 mL N,N-dimethylformamide (DMF) and then sonicated for 1 h to make it dispersed evenly. Then Ni(NO_3_)_2_∙6H_2_O (116.4 mg, 0.4 mmol) and 2-methylimidazole (32.8 mg, 0.4 mmol) were added into the above solution. After vigorous stirring for 30 min, the solution was transferred into a Teflon-lined autoclave with capacity of 23 mL and put into an oven at 85 °C for 72 h. After the autoclave was cooled to room temperature naturally, the product was collected and washed with methanol for several times. Finally, the product was dried in the vacuum at 60 °C for 6 h (Fig. 1, Fig. 2).

**Fig. 3:**
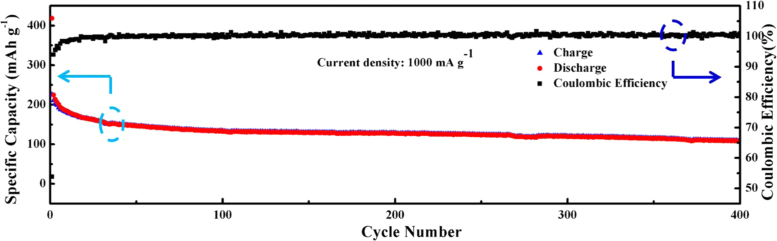
Electrochemical performance of Ni_2_P/NG/Ni_2_P composite.

### 2.2 Synthesis of Ni_2_P/NG/Ni_2_P nanoarrays

Ni_2_P/RGO/Ni_2_P was prepared via two steps. At first, Ni-based precursor/GO/Ni-based precursor was calcined at 450 °C in argon atmosphere for 2 h to obtain NiO/NG/NiO. Then NiO/NG/NiO and appropriate amount of NaH_2_PO_2_ powders were put into two separate positions in one closed combustion boat and then heated at 300 °C with a temperature increasing speed of 3 °C min^−1^ in argon atmosphere for 2 h.
