# Supplementary material for: Sandwich-like Ni2P nanoarray/nitrogen-doped graphene nanoarchitecture as a high-performance anode for sodium and lithium ion batteries
Source: Data Brief. 2018 Aug 30;20:1999–2002. doi: 10.1016/j.dib.2018.08.158 (PMC6172565; doi:10.1016/j.dib.2018.08.158)
Supplement: Supplementary file 1 — Supplementary material [file mmc1.docx]

"Conflict of Interest

All the authors confirm as No conflict of Interest"
